# Genetic structure among morphotypes of the endangered Brazilian palm *Euterpe edulis* Mart (Arecaceae)

**DOI:** 10.1002/ece3.6348

**Published:** 2020-05-19

**Authors:** Gislaine Mendes Coelho, Alesandro Souza Santos, Ivandilson Pessoa Pinto de Menezes, Roberto Tarazi, Fernanda Maria Oliveira Souza, Maria das Graças Conceição Parada Costa Silva, Fernanda Amato Gaiotto

**Affiliations:** ^1^ Departamento de Ciências Biológicas Centro de Biotecnologia e Genética Universidade Estadual de Santa Cruz Ilhéus Brazil; ^2^ Laboratório de Ecologia Aplicada à Conservação Universidade Estadual de Santa Cruz Ilhéus Brazil; ^3^ Laboratório de Genética e Biologia Molecular Núcleo de Ciências Biológicas Instituto Federal Goiano Urutaí Brazil; ^4^ BASF Discovery Breeder Trindade Brazil; ^5^ Centro de Pesquisa do Cacau (CEPEC) Comissão Executiva do Plano da Lavoura Cacaueira (CEPLAC) Ilhéus Brazil

**Keywords:** ecotypes, genetic bottleneck, palm, population genetics, tropical rainforest

## Abstract

*Euterpe edulis* (Arecaceae) Mart has high ecological and economic importance providing food resources for more than 58 species of birds and 20 species of mammals, including humans. *E*. *edulis* is the second most exploited nontimber product from Brazilian Atlantic Forest. Due to overexploitation and destruction of habitats, *E. edulis* is threatened by extinction. *Euterpe edulis* populations have large morphological variations, with individuals having green, red, or yellow leaf sheath. However, no study has related phenotypic distinctions between populations and their levels of genetic structure. Thus, this study aimed to evaluate the diversity and genetic structure of different *E. edulis* morphotypes. We sampled 250 adult individuals in eight populations with the different morphotypes. Using 14 microsatellite markers, we access genetic diversity through population genetic parameters calculated in the GenAlex program and the diveRsity package in R. We used the Wilcoxon test to verify population bottlenecks and the genetic distance of Nei and Bayesian analysis for genetic clusters. The eight populations showed low allele richness, low observed heterozygosity, and high inbreeding values (*f*). In addition, six of the eight populations experienced genetic bottlenecks, which would partly explain the low genetic diversity in populations. Cluster analysis identified two clusters (*K* = 2), with green morphotype genetically distinguishing from yellow and red morphotypes. Thus, we show, for the first time, a strong genetic structure among *E. edulis* morphotypes even for geographically close populations.

## Graphical abstract

We recorded low genetic variability and genetic bottleneck effect in *Euterpe edulis* populations, which also have unusual genetic structure for populations of this species. In addition, the evaluated populations formed two genetic clusters, separating typical populations (green morphotype) from populations with yellow or red morphotypes that have restricted occurrence within the Brazilian Atlantic Forest. Thus, we showed, for the first time, that morphologically distinct populations of *E. edulis* have a strong genetic structure, as a possible response to local environmental factors.

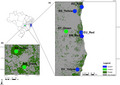

## 1 INTRODUCTION

Arecaceae is the family of Palms and has pantropical distribution, with about 183 genera and 2,400 species (Dransfield et al., 2008). Arecaceae species are key components of forest structuring and are also important because they provide fruits and seeds that maintain a high richness of frugivores in these environments (Benchimol et al., 2016; Elena et al., 2014; Galetti et al., 2013). In Brazil, 37 genera and 297 species of Arecaceae are found, being approximately 46% of these endemic species (Leitman, Soares, Henderson, Noblick, & Martins, 2015). Studies show that this high percentage of endemism is probably related to environmental characteristics, such as soil fertility and climatic factors (Eiserhardt, Svenning, Kissling, & Balslev, 2011; Salm, De Salles, Alonso, & Schuck‐Paim, 2007). For the Brazilian Atlantic Forest, 61 Arecaceae species are found, which are in constant risk of being lost due to the exacerbated loss of their natural habitat (Benchimol et al., 2016; Leitman et al., 2015).

*Euterpe edulis* Mart (Arecaceae) has a great ecological and economic relevance (Carvalho, Galetti, Colevatti, & Jordano, 2016; Elena et al., 2014; Galetti et al., 2013; Reis et al., 2000). In Brazil, this species is primarily widely distributed in the Atlantic Forest but also occurs in the Gallery Forests of the Cerrado (Leitman et al., 2015). *Euterpe edulis* seeds and fruits are important food resources for about 58 bird species and 20 mammal species playing an important ecological role in maintaining the diversity of frugivores in the Atlantic Forest (Galetti et al., 2013). In economic terms, the species is the second most exploited nontimber product of the Atlantic Forest Brazilian, through the extraction of its apical meristem called heart‐of‐palm for human consumption (Pizo & Vieira, 2004; Reis et al., 2000; Silva Matos, Freckleton, & Watkinson, 1999). The illegal harvesting of heart‐of‐palm causes the death of individuals, which is a serious problem, since the species does not sprout or have tillering. Therefore, illegal harvesting is the primary cause leading to the species listing as endangered flora in Brazil (Leitman et al., 2015). In addition to the negative impacts of predatory harvesting, the reduction and fragmentation of the Atlantic Forest further threaten this species (Carvalho, Ribeiro, Côrtes, Galetti, & Collevatti, 2015; Fleury & Galetti, 2006; Santos, Cazetta, Dodonov, Faria, & Gaiotto, 2016).

*Euterpe edulis* is a monoecious species with predominance of cross‐pollination, performed mainly by small bees (e.g., *Trigona spinipes*) and seeds dispersed mainly by birds (Gaiotto, Grattapaglia, & Vencovsky, 2003; Galetti et al., 2013; Reis et al., 2000). The populations of *E. edulis* may present large morphological variations, as for example, leaf sheath color and number of rachila, besides distinct demographic characteristics along their area of occurrence. These characteristics can influence different ecological aspects, such as fruit production, with a potential influence on the recruitment of new individuals in the population (Bovi, Godoy, & Saes, 1987; Mantovani & Morellato, 2000; Reis, Kageyama, Reis, & Fantini, 1996; Silva, Martini, & De Araújo, 2009). Motivated by these differences, some scientists have proposed separation into two species and one variety: Populations with green sheath individuals as *E. edulis*; those with red sheaths as *E. spiritosantensis*; and yellow sheath individuals as *E. edulis var. clausa* (Bovi et al., 1987; Leitman et al., 2015; Mantovani & Morellato, 2000; Reis et al., 1996; Silva et al., 2009). Despite of the morphological and demographic differences being are well known and described in literature, *E. edulis* is the only taxonomically accepted name with the others considered synonyms (Leitman et al., 2015).

There is a shortage of genetic studies that associate the phenotypic distinction between natural populations with genetic structure. Currently, it is only known that populations of *E. edulis* in different environmental conditions are genetically different (Alves‐Pereira et al., 2019; Brancalion et al., 2018). However, it is not known whether populations with morphological differences are also genetically different. The central objective of this study is to evaluate the genetic diversity and to verify whether there is genetic differentiation between the different *E. edulis* morphotypes.

## 2 MATERIAL AND METHODS

### 2.1 Sampling areas

**FIGURE 1:**
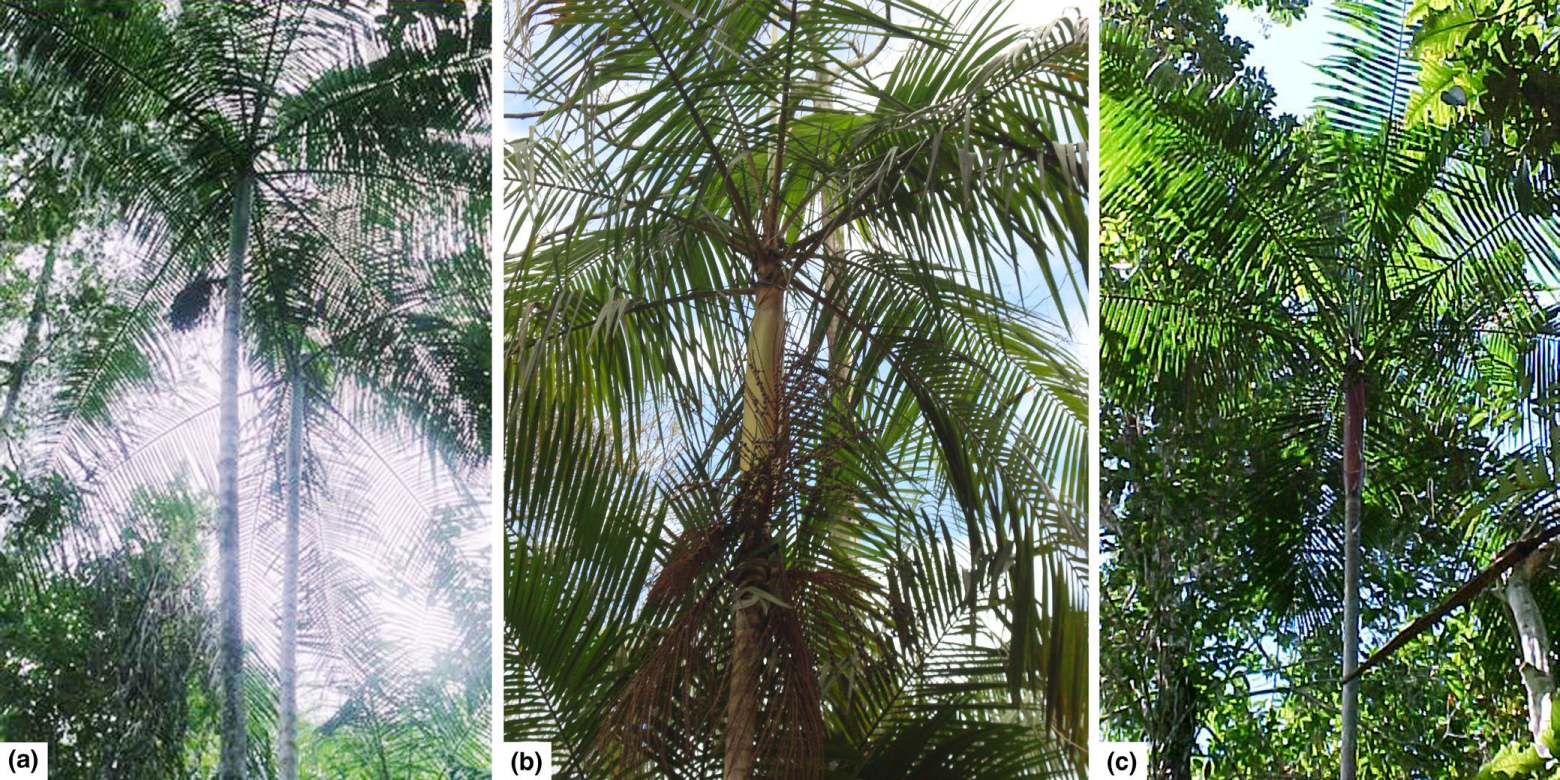
Phenotypic distinction between the (a) green (b) yellow and (c) red morphotypes found in the populations of *E. edulis*

**FIGURE 2:**
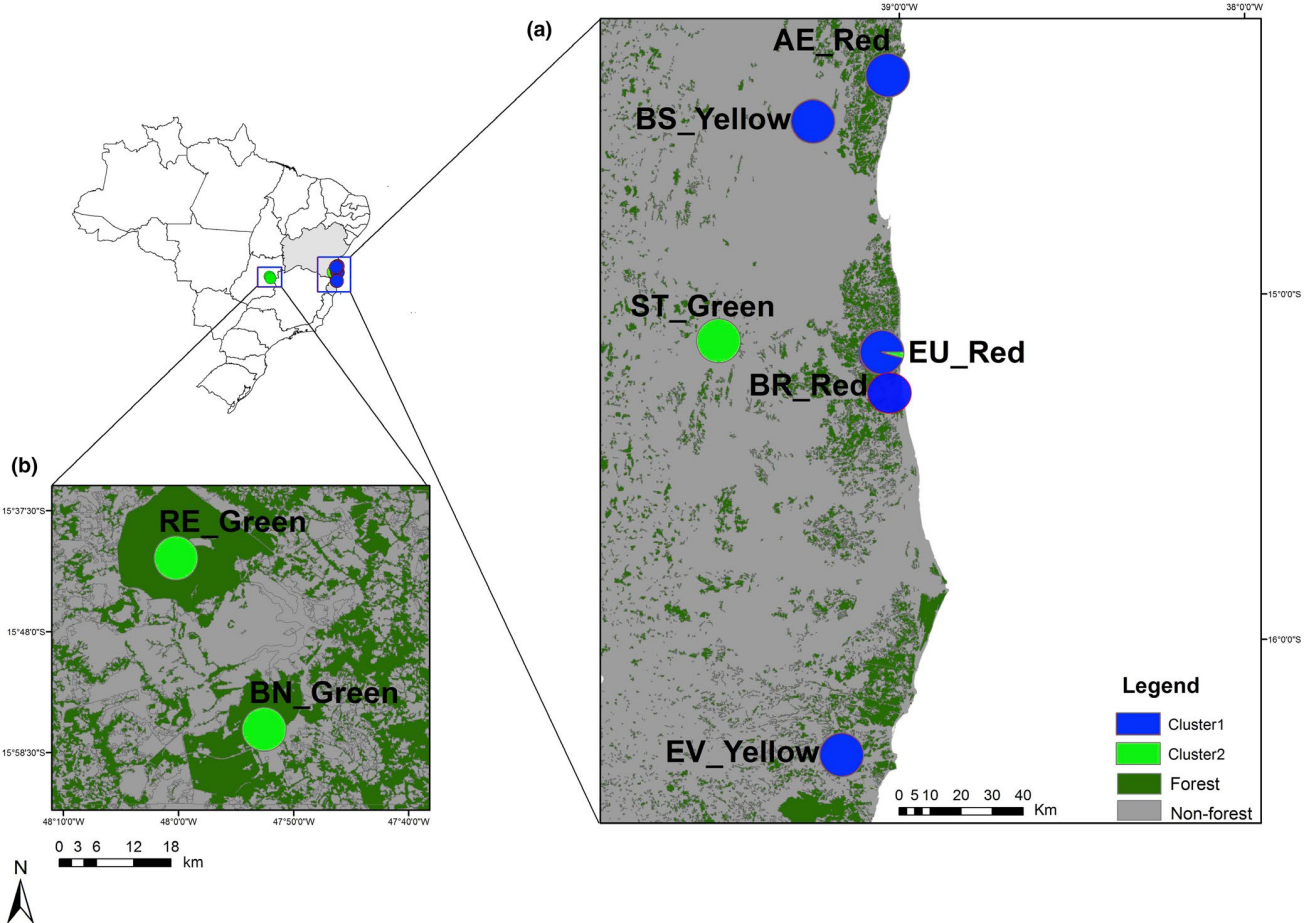
Map of Brazil, highlighting the state of Bahia and the Distrito Federal, indicating the region of the study with the current forest cover. (a) Populations of *E. edulis* sampled in Bahia and (b) Populations of *E. edulis* analyzed in Distrito Federal. Populations colors (blue or green) indicate the grouping (Δ*K* = 2) assigned in the Structure software. Map data: Atlas of the Atlantic Forest remnants of the year 2016 obtained from SOS Mata Atlântica (https://www.sosma.org.br) and State Geoinformation System—SIEG of the state of Goiás (http://www2.sieg.go.gov.br/). AE, Farm Alto da Esperança; BS, Farm Boa Sorte; BN, Brasilia National Park; BR, Una Biological Reserve; EV, Private Reserve of Natural Patrimony Estação Veracel; EU, Ecoparque de Una; RE, Roncador Ecological Reserve; ST, Private Reserve of Natural Patrimony Serra do Teimoso. The population morphotype is indicated by the names yellow, red, or green

The populations studied were chosen based on their distinct morphotypes (Figure 1 and Table 1) and geographical distances (Figure 2). Eight populations were sampled; two in gallery forests from the *Cerrado* biome (Brazilian savanna in Distrito Federal—DF, central west of Brazil) and six from the Atlantic Forest (Bahia, northeastern of Brazil; Figure 2). We did not observe individuals belonging to different morphotypes within the populations.

**TABLE 1:** *Euterpe edulis* populations sampled with their respective morphotypes, sample size with estimation of genetic parameters and Wilcoxon sign‐rank test

Population	Morphotype	*N*	PA	AR	*H*_O_	*H*_E_	*f* (CI 95%)	IAM	TPM	SMM
EV	Yellow	16	1.07	4.61	0.44	0.62	0.28 (0.16–0.39)	0.076	0.891	0.979
BS	Yellow	40	0.57	5.62	0.50	0.70	0.28 (0.20–0.36)	0.020	0.749	0.982
BR	Red	40	0.64	5.17	0.55	0.68	0.19 (0.12–0.26)	0.291	0.979	0.991
AE	Red	40	1.35	5.69	0.54	0.75	0.28 (0.23–0.33)	0.000	0.786	0.948
EU	Red	33	0.86	5.28	0.51	0.67	0.24 (0.17–0.29)	0.010	0.786	0.987
ST	Green	20	0.78	4.46	0.39	0.61	0.36 (0.27–0.45)	0.039	0.891	0.991
BN	Green	28	0.71	5.69	0.71	0.75	0.05 (−0.01–0.11)	0.002	0.913	0.985
RE	Green	33	1.00	5.94	0.66	0.75	0.11 (0.04–0.017)	0.052	0.985	0.998
Mean	—	**31.25 (±8.69)**	**0.87 (±0.27)**	**5.31 (±0.50)**	**0.53 (±0.21)**	**0.69 (±0.14)**	**0.22 (0.15–0.29)**	—	—	—

Note

*p* values Significant are shown in bold.

Abbreviations: ( ) = standard deviation; AE, Farm Alto da Esperança; AR, Allelic richness; BN, Brasilia National Park; BS, Boa Sorte Farm; BR, Una Biological Reserve; EU, Ecoparque de Una; ST, Private Reserve of Natural Patrimony Serra do Teimoso; EV, Private Reserve of Natural Patrimony Estação Veracel; *f*, Inbreeding coefficient; *H*_E_, expected heterozygosity; *H*_O_, Observed heterozygosity; IAM, infinite alleles model; *N*, Size samples; PA, Mean private alleles; RE, Roncador Ecological Reserve; SMM, stepwise mutation model; TPM, two‐phase model.

The two populations sampled in DF are located in gallery forests, which are a type of perennial forest vegetation immersed in savanna formations, which accompany small streams and usually have nutrient‐rich soils (Haridasan, 1998). The DF populations occur within protected areas, namely the Brasilia National Park (PNB) and the Roncador Ecological Reserve (IBGE), which are located 10 km and 20 km from Brasilia‐DF, respectively.

The sampled populations in Bahia are located in the region with the largest remnants of the Atlantic Forest of northeastern Brazil (Ribeiro, Metzger, Martensen, Ponzoni, & Hirota, 2009) and are considered a priority for conservation due to high biodiversity of this region (Martini, Fiaschi, Amorim, & Paixão, 2007). Of these populations, four are located in protected areas and two in unprotected areas. Populations in protected areas include the following: (1) the Private Reserve of Natural Patrimony Serra do Teimoso (ST) located in the municipality of Jussari, transitionary forest between the rainforest at the tops of hills and semideciduous forest at the base; (2–3) rainforest population of Ecoparque de Una (EU) and the Una Biological Reserve (BR), located in the municipality of Una (Thomas, Carvalho, Amorim, Garrison, & Arbelaez, 1998); and (4) the Private Reserve of Natural Patrimony Estação Veracel (EV), which is located in the rainforests of the municipality of Eunápolis. The remaining two populations are not afforded protection in conservation estate but occur in the rainforests of the Atlantic Forests on Alto da Esperança Farm (AE) in the municipality of Itacaré and on Boa Sorte Farm (BS) in the municipality of Uruçuca.

### 2.2 Microsatellite marker analysis

In total, samples were collected from the root tissue of 250 adult individuals through active search (Table 1), in order to cover as much of each area as possible, avoiding the sampling of individuals geographically close to each other, aiming to sample the genetic pool of each collection area. Posteriorly, the DNA was obtained according to Doyle and Doyle (1987), and the genetic material was amplified with a total of 14 nuclear microsatellite fluorescent primer pairs (*EE2/EE5/EE23/EE32/EE9/EE25/EE43/EE45/EE47/EE48/EE52/EE54/EE59/EE63*) developed by Gaiotto, Brondani, and Grattapaglia (2001).

The amplification reactions were performed by PCR in a final volume of 13 µl containing 7.5 ng of genomic DNA, 1× buffer (10 mmol/L Tris‐HCl, 50 mmol/L KCl and 21.5 mmol/L MgCl, pH 8.3), 0.27 µmol/L of each primer, 2.0 mmol/L MgCl_2_, 0.25 mg/ml BSA, 0.25 mmol/L dNTP, and 1U of Taq DNA polymerase in ultrapure sterile water. The PCRs were performed under the following conditions: 96°C for 2 min; 34 cycles of 94°C for 1 min, primer‐specific annealing as per Gaiotto et al. (2001, T°C) for 1 min, 72°C for 1 min; with a final elongation performed at 72°C for 7 min. The analysis of amplicons in a denaturing gel (7 mol/L urea) with 4% polyacrylamide (Long Ranger 50%—Cambrex) was performed in a multiplex system, in a semiautomatic ABI 377 sequencer (Applied Biosystems) by using virtual filter D.

### 2.3 Data analysis

To estimate the frequency of null alleles and to identify possible genotyping errors, we use the MICRO‐CHECKER 2.2.3 program (Van Oosterhout, Utchinson, Wills, & Shipley, 2004). The populations presented low average frequency of null alleles (≤0.08), not compromising our results. The corrected genotypes from MICRO‐CHECKER were used for the genetic analysis, ensuring greater accuracy in the results. After, to test the linkage disequilibrium between all pairs of loci in each population, we use the FSTAT 2.9.3 program (Goudet, 2001) and to calculate the Hardy–Weinberg equilibrium at each locus within each population in the GenAlex 6.5 program (Peakall & Smouse, 2012).

The genetic diversity of *E. edulis* morphotypes was estimated using different population genetics parameters calculated by GenAlex 6.5 (Peakall & Smouse, 2012), FSTAT 2.9.3 (Goudet, 2001), and diveRsity package in the R software (Keenan, McGinnity, Cross, Crozier, & Prodohl, 2013). In the GenAlex, we calculated the mean number of private alleles (PA). In the FSTAT, we calculate rarified allelic richness (AR), given there is some uneven sampling among populations under analysis. Additionally, in diveRsity package, we used the divBasic function to calculate the expected heterozygosity (*H*_E_), observed heterozygosity (*H*_O_), and inbreeding coefficient (*f*). To consider the 95% confidence interval of *f* and the standard deviation for *H*_E_ and *H*_O_, we calculated 10,000 bootstraps which the divBasic function.

To identify population bottenecks, the Wilcoxon test in the software BOTTLENECK version 1.2.02 02 (Cornuet & Luikart, 1996) was used to test whether populations have excess heterozygosity (Piry, Luikart, & Cornuet, 1999). The Wilcoxon test is recommended because of its power to detect population bottleneck when few molecular markers (<20 loci) are used, as is the case in our study. As microsatellites can present different mutational models, we performed the Wilcoxon test considering the infinite allele model (IAM), stepwise mutation model (SMM), and two‐phase model (TPM; Piry et al., 1999). For the TPM model, the proportion of SMM in TPM = 0.000, and variance of the geometric distribution for TPM = 0.36, which generally represent the most sensitive values for microsatellite markers (Piry et al., 1999).

To estimate the level of genetic structure among the populations, we calculated the observed *F*_ST_ values and the 95% confidence interval with 10,000 bootstraps, using the diffCalc function of the diveRsity package in software R (Keenan et al., 2013; http://www.r‐project.org/). Subsequently, to assess whether geographical distance (km) influenced in *F*_ST_ values obtained between populations, we performed a Mantel test (Mantel, 1967) using the ecodist package (Goslee & Urban, 2007) in the software R (http://www.r‐project.org/).

To evaluate the population clustering, we calculated the genetic distance from Nei in GenAlex 6.5 (Peakall & Smouse, 2012) and the Bayesian clustering in Structure program (Evanno, Regnaut, & Goudet, 2005). Using Nei's genetic distance, we performed a heatmap analysis with the eight populations of *E. edulis* in heatmaply package (Galili, O’Callaghan, Sidi, & Sievert, 2017) in software R. To determine the number of groups in the heatmap, we used the hierarchical clustering algorithm with the Euclidean distance and the average method, implemented in heatmaply package (Galili et al., 2017) in software R. The settings of the Bayesian model inferred automatically the number of distinct genetic groups according to mixed model that was often correlated. An analysis of the number of populations (*K*) was performed for values ranging from 1 to 10 with ten independent chains, each chain with a burn‐in length of 50,000 iterations, followed by 100,000 repetitions of the MCMC (Markov and Monte Carlo chain). The Δ*K* (actual number of groups) was determined on the basis of the average values of *L* (*K*) as produced for ten repetitions for each *K*, according to the method of Evanno et al. (2005), using Structure Harvester program (Earl and VonHoldt, 2012).

## 3 RESULTS

### 3.1 Genetic diversity and population bottlenecks

Our results did not show evidence of genotypic linkage disequilibrium between loci (results not shown). However, only 36% in average of the 14 loci used present expected genotypic frequencies at Hardy–Weinberg equilibrium (Table S1).

The eight populations of *E. edulis* showed low to moderate allelic richness (Mean = 5.31 ± 0.50), and all populations had private alleles (Table 1). The observed heterozygosity (*H*_O_) was low to moderate (Mean = 0.53 ± 0.21), except for BN and RE with high values (Table 1). The expected heterozygosity (*H*_E_) was moderate to high (Mean = 0.69 ± 0 0.14), with emphasis for AE, BN, and RE (Table 1). The values of inbreeding coefficient (*f*) were high (Mean = 0.22, CI = 0.15–0.29), except for BN and RE, although no values are nonzero (Table 1).

The different mutational models (IAM, TPM, and SMM) used in the Wilcoxon test for population bottlenecks showed divergent results. The IAM model detected significant excess of heterozygosity for the BS, AE, EU, ST, BN, and RE populations, while the SMM and TPM models did not detect genetic bottlenecks in any of the eight *E. edulis* populations evaluated (Table 1).

### 3.2 Genetic structure

The analysis of the genetic structure with *F*_ST_ revealed that the populations have moderate to high genetic differentiation, though this is lessor for BN‐RE populations (Table 2). Although all *F*_ST_ values are moderate or high, they are within the confidence interval and do not differ statistically from zero (Table 2). The Mantel test showed that the geographical distance does not correlate significantly with the pattern of genetic differentiation found between populations (*r* = .13, *p* = .33, Figure S1).

**TABLE 2:** Matrix between geographical distance and *F*_ST_

	AE (Red)	BS (Yellow)	BR (Red)	EV (Yellow)	EU (Red)	ST (Green)	BN (Green)	RE (Green)
AE (Red)	—	0.12 (0.10–0.14)	0.14 (0.12–0.16)	0.21(0.18–0.25)	0.14 (0.11–0.17)	0.22 (0.20–0.25)	0.17 (0.15–0.19)	0.19 (0.16–0.21)
BS (Yellow)	28	—	0.16 (0.13–0.18)	0.18 (0.15–0.22)	0.18 (0.16–0.21)	0.27 (0.25–0.30)	0.22 (0.20–0.24)	0.22 (0.20–0.24)
BR (Red)	89	76	—	0.24 (0.21–0.28)	0.17 (0.15–0. 20)	0.28 (0.25–0.31)	0.22 (0.20–0.24)	0.23 (0.21–0.25)
EV (Yellow)	218	203	130	—	0.28 (0.25–0.32)	0.33 (0.30–0.37)	0.25 (0.23–0.29)	0.26 (0.23–0.29)
EU (Red)	88	76	2	129	—	0.25 (0.21–0.28)	0.18 (0.16–0.20)	0.22 (0.20–0.24)
ST (Green)	100	75	49	138	51	—	0.20 (0.18–0.23)	0.23 (0.20–0.26)
BN (Green)	968	941	953	937	959	910	—	0.07 (0.06–0.09)
RE (Green)	967	939	952	916	949	903	31	—

Note

Diagonally upper observed values estimated *F*_ST_. Diagonally lower observed values distances (km) between the sampled populations.

Abbreviations: ( ) = 95% confidence interval; AE, Farm Alto da Esperança; BN, Brasilia National Park; BR, Una Biological Reserve; BS, Boa Sorte Farm; EU, Ecoparque de Una; EV, Private Reserve of Natural Patrimony Estação Veracel; RE, Roncador Ecological Reserve; ST, Private Reserve of Natural Patrimony Serra do Teimoso.

### 3.3 Population clustering

**FIGURE 3:**
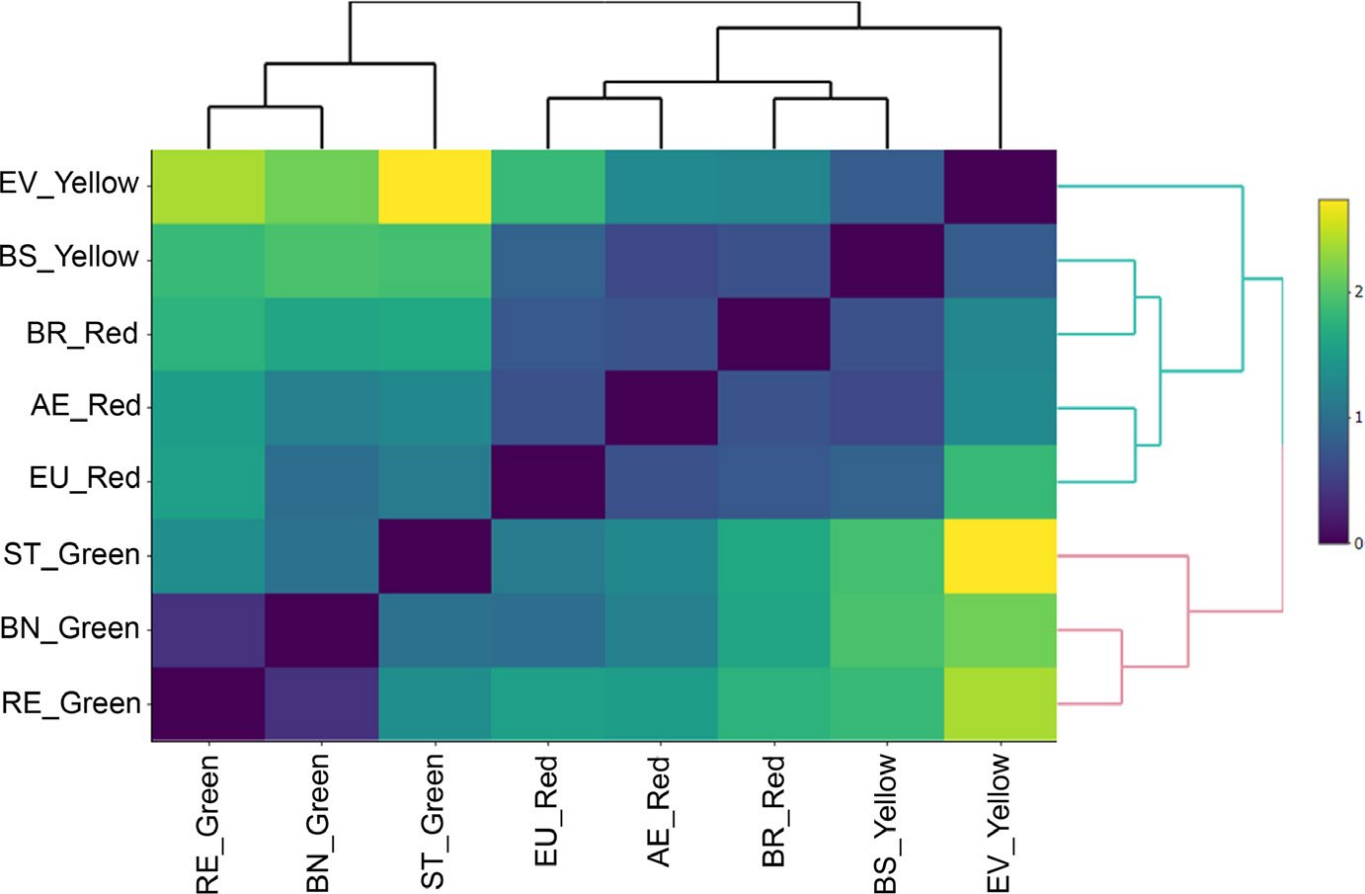
Heatmap of the eight populations of *E. edulis* with Euclidean distance and clust average method using the distance of Nei (1978), indicating the two groups established (group 1 blue lines and group 2 with pink lines). AE, Farm Alto da Esperança; BN, Brasilia National Park; BS, Farm Boa Sorte; BR, Una Biological Reserve; EV, Private Reserve of Natural Patrimony Estação Veracel; EU, Ecoparque de Una; RE, Roncador Ecological Reserve; ST, Private Reserve of Natural Patrimony Serra do Teimoso. The population morphotype is indicated by the names yellow, red, and green

The heatmap with clustering using the Nei's genetic distance showed that the eight sampled populations form two clusters (Figure 3 and Figure S2). The populations with green morphotypes which were sampled in the Distrito Federal (BN and RE) and southern Bahia (ST) clustered separately from the red (EU, AE, and BR) and yellow (BS and EV) morphotypes sampled in southern Bahia (Figure 3).

**FIGURE 4:**
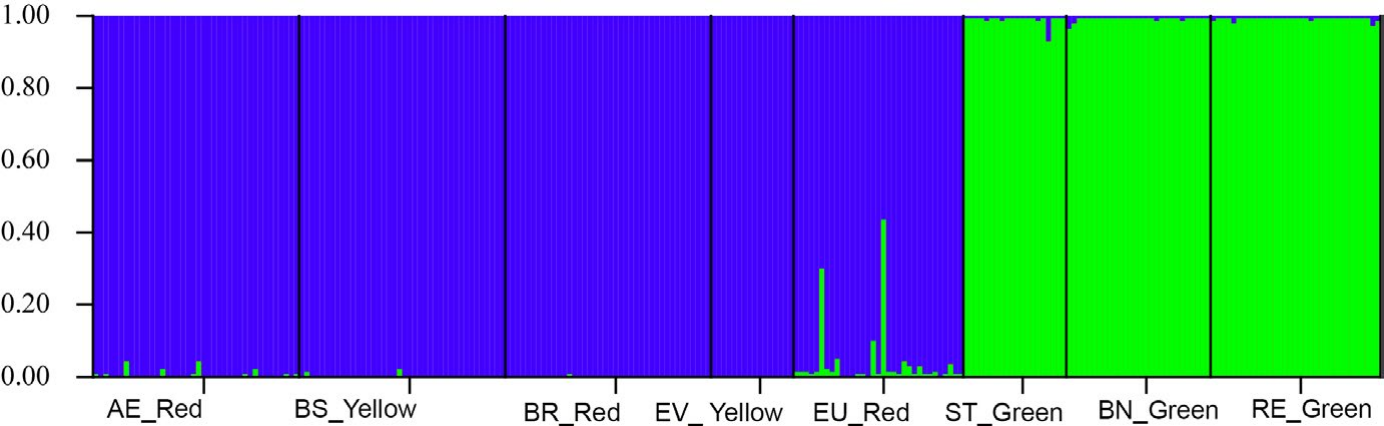
STRUCTURE bar plots for *E. edulis* sampled in eight populations. Each sampled individual of *E. edulis* is represented by a single vertical line with different colors (green and blue) representing assignment probabilities to the two inferred genetic clusters (Δ*K* = 2). AE, Farm Alto da Esperança; BS, Farm Boa Sorte; BN, Brasilia National Park; BR, Una Biological Reserve; EV, Private Reserve of Natural Patrimony Estação Veracel; EU, Ecoparque de Una; RE, Roncador Ecological Reserve; ST, Private Reserve of Natural Patrimony Serra do Teimoso. The population morphotype is indicated by the names yellow, red, or green

The Bayesian clustering method estimated the true number of groups for the eight populations as *K* = 2 (Figure 4 and Figure S3). On average, the shared ancestry among the *E. edulis* palm was 98.8% and 99.4% in the groups marked in blue and green, respectively. The blue group was composed of all individuals sampled in the EU, AE, BR, BS, and EV populations in southern Bahia that have red or yellow morphotypes (Figure 1). The green group contains all the individuals sampled in the RE and BN populations in Distrito Federal and ST in southern Bahia that have green morphotype (Figure 1).

## 4 DISCUSSION

In the present study, we recorded low genetic variability and genetic bottleneck effect in *Euterpe edulis* populations, which also have unusual genetic structure for populations of this species (Gaiotto et al., 2003; Santos et al., 2015). In addition, the evaluated populations formed two genetic clusters, separating typical populations (green morphotype) from populations with yellow or red morphotypes that have restricted occurrence within the Brazilian Atlantic Forest. Thus, we showed, for the first time, that morphologically distinct populations of *E. edulis* have a strong genetic structure, as a possible response to local environmental factors.

### 4.1 Genetic diversity and population bottlenecks

In general, there is a low genetic diversity in the eight populations of *E. edulis* when considering the richness of alleles compared to other populations of the species, using microsatellite markers (Carvalho et al., 2015, 2017; Conte, Sedrez Dos Reis, Mantovani, & Vencovsky, 2008). In theory, several ecological or anthropogenic factors can negatively impact genetic diversity, such as the reduction or inefficiency of dispersers and pollinators, reduction in effective population size, causes related to habitat loss and fragmentation, or even illegal extraction of species (Browne, Ottewell, & Karubian, 2015; Carvalho et al., 2016; Chung et al., 2014; da Carvalho et al., 2017; Ellegren & Galtier, 2016; Young, Boyle, & Brown, 1996). Even in this context, all populations had a considerable proportion of private alleles, demonstrating the importance of these areas for the conservation of this endangered species.

Considering the observed heterogeneity (*H*_O_), six populations have low or moderate values and lower than those reported in other populations of the species (Carvalho et al., 2015). In addition, expected heterozygosity (*H*_E_) values are moderate to high and always higher than *H*_O_ values in all populations, indicating deviation from that expected under drift–mutation equilibrium (see Table S1) in an idealized, panmictic population (in the absence of evolutionary forces). As a consequence, all populations have positive and high values of inbreeding (*f*), indicating nonrandom mating occurring, which may cause reduction of genetic diversity (Xue et al., 2015). In this scenario, the genetic erosion may arise and even increase over generations, as individuals would become more related to each other as result of nonrandom mating within populations (Xue et al., 2015). On the other hand, the populations RE and BN have the highest genetic diversity (*H*_O_, *H*_E_) values and the lowest inbreeding measures, indicating the best genetic conservation status among the studied populations. These values are probably influenced by the occurrence of gene flow between RE and BN, which may favor the maintenance of genetic variability over time (Gaiotto et al., 2003; Santos et al., 2016).

The Wilcoxon test, with the infinite allele mutational model (IAM), revealed that six of the eight populations studied have recently experienced genetic bottlenecks. However, the two‐phase model (TPM) and stepwise mutation model (SMM) had divergent results, not detecting genetic bottleneck in any of the evaluated populations. This result was expected to some extent because, although microsatellite markers may adhere to different mutational models (AIM, TPM, or SMM), genetic bottlenecks have been reported mainly for the AIM model (Piry et al., 1999; Santos et al., 2019). The genetic bottleneck or excess of H_E_ occurs when the population has experienced a recent reduction in effective population size, causing a reduction in the number of alleles faster than in *H*_E_ (Cornuet & Luikart, 1996; Piry et al., 1999). The genetic bottleneck identified in most of the populations evaluated in this study would explain the low allelic richness. Even if some population did not go through a genetic bottleneck (e.g., BR and EV), there is evidence of genetic structure (Figures 3 and 4), which could potentially lead to nonrandom crossing within these areas due to isolation (Mosca, González‐Martíınez, & Neale, 2014; Sexton, Hangartner, & Hoffmann, 2014). Thus, we believe that both the genetic bottleneck and the degree of isolation may explain the reduction of genetic diversity in terms of richness of alleles, *H*_O_ < *H*_E_, high values of *f,* and presence of private alleles in the populations.

### 4.2 Genetic structure

Considering all populations, *F*_ST_ values were generally moderate to high, indicating a limited gene flow between populations. These *F*_ST_ values indicate unusual genetic divergence among *E. edulis* populations compared to other populations of the species, even when under influence of fragmentation and geographically distant (Conte et al., 2008; Santos et al., 2015). Thus, we believe that the differences found between the populations of this palm tree should not be related to habitat fragmentation, but to naturally occurring evolutionary events, such as local adaptation (Brancalion et al., 2018). In addition, it is important to draw attention to RE and BN that have the same morphotype and are geographically close, with low to moderate *F*_ST_, indicating occurrence of gene flow, as reported by Gaiotto et al. (2003) for these same populations. However, it is important to note that, although *F*_ST_ values were high in most cases, all values are within the confidence interval and are not statistically different from zero.

The pattern of genetic differentiation (*F*_ST_) is not explained by geographical distance as theoretically expected, in which geographically closer populations are genetically more similar (Diniz‐filho et al., 2013; Ramírez‐Barrera, Velasco, Orozco‐Téllez, Vázquez‐López, & Hernández‐Baños, 2019; Wright, 1943). Thus, we emphasize that the pattern of genetic differentiation found between populations is probably influenced mainly by morphological differences, as demonstrated in the cluster analysis.

### 4.3 Population clustering

Although *F*_ST_ values were moderate to high between the sampled populations, clustering using Nei genetic distance and Bayesian analysis identified two genetic groups. In the Bayesian approach, *F*_ST_ value greater than 0.20 can allow for more accurate cluster identification and correctly attribute individuals to a true cluster (Latch, Dharmarajan, Glaubitz, & OER, 2006). Thus, the reported *F*_ST_ values probably contributed to a greater inference power of the genetic clusters.

The grouping into two clusters (Figures 3 and 4) indicates a genetic ancestry between populations with green morphotypes and that it is genetically distinguished from yellow and red morphotypes. Thus, our results are the first to show that there is genetic divergence within morphotypes of *E. edulis* rather than phenotypic plasticity, possibly due to a response in environmental or ecological differences (Funk & Murphy, 2010). The grouping of the yellow morphotype with the red indicates that this morphological differentiation is probably more recent compared to the green morphotype. In this context, considering that there are proposals in the literature to taxonomically separate the morphotypes: green sheath as *Euterpe edulis*; red sheaths as *E. spiritosantensis;* and yellow sheaths as *E. edulis var. clausa*. Our result of genetic grouping supports only the separation of the green morphotype (*E. edulis*) from the others. However, complementary genetic studies (e.g., crossover experiment between morphotypes) should be performed to test this taxonomic separation.

Although we have used microsatellite markers that are considered neutral, we believe that the genetic clusters that distinguish *E. edulis* morphotypes are a consequence of populations being shaped in different environmental or ecological conditions. This hypothesis is based on recent studies using SNPs (single‐nucleotide polymorphism) markers, demonstrating that *E. edulis* populations inserted in different environmental and ecological conditions are genetically different (Alves‐Pereira et al., 2019; Brancalion et al., 2018). Thus, it is believed that there is adaptation of species to different conditions and that this adaptation remains mainly local, favoring the genetic divergence directed by the environment (Rellstab et al., 2017; Sork, 2017). Future studies could include measuring local environmental variables of populations sampled and the use of makers under selection (e.g., SNPs) to test our hypothesis of local adaptation with morphological and genetic differentiation.

## 5 CONCLUSION

The genetic grouping of yellow and red morphotypes that are only restricted to the state of Bahia and Espírito Santo in Brazil is genetically different from the green morphotype of *E. edulis* that have occurrence areas in across the entire distribution of the species. The relationship between phenotypic and genetic variation is an important advance in scientific knowledge for future conservation measures for this endangered species. In consideration of the low genetic variability reported in this study, the development of conservation plans is highly recommended, particularly for populations with yellow or red morphotypes that have limited geographical occurrence.

## CONFLICT OF INTERESTS

The authors declare no conflict of interest.

## AUTHOR CONTRIBUTION

**Gislaine Mendes Coelho:** Conceptualization (equal); Investigation (lead); Methodology (lead); Validation (lead); Visualization (supporting); Writing‐original draft (supporting). **Alesandro Souza Santos:** Data curation (lead); Formal analysis (lead); Writing‐original draft (lead); Writing‐review & editing (lead). **Ivandilson Pessoa Pinto de Menezes:** Conceptualization (supporting); Writing‐original draft (supporting). **Roberto Tarazi:** Conceptualization (equal); Writing‐original draft (equal). **Fernanda Maria Oliveira Souza:** Conceptualization (supporting); Writing‐original draft (supporting). **Maria das Graças Conceição Parada Costa Silva:** Conceptualization (supporting); Writing‐original draft (supporting). **Fernanda Amato Gaiotto:** Conceptualization (lead); Funding acquisition (lead); Investigation (lead); Methodology (lead); Project administration (lead); Resources (lead); Supervision (lead); Writing‐original draft (equal).

## Supporting information

Supplementary Material

## Data Availability

Relationship between pairwise F_ST_ and geographical distances **(**Figure S1), estimated number of groups using the average method (Figure S2), variation of the second order of the average values of maximum likelihood (Figure S3) uploaded as online Supplementary material. In addition, the raw data were deposited in the Dryad: https://doi.org/10.5061/dryad.547d7wm5g.
