# Supplementary material for: Genetic structure among morphotypes of the endangered Brazilian palm Euterpe edulis Mart (Arecaceae)
Source: Ecol Evol. 2020 May 19;10(12):6039–48. doi: 10.1002/ece3.6348 (PMC7319139; doi:10.1002/ece3.6348)
Supplement: Supplementary file 1 — Supplementary Material [file ECE3-10-6039-s001.docx]

**APPENDIX**

**Table 1.** Summary of tests for Hardy-Weinberg Equilibrium for the 14 loci within the eight populations of *E. edulis*.

| Population | Morphotype | Loco1 | Loco2 | Loco3 | Loco4 | Loco5 | Loco6 | Loco7 | Loco8 | Loco9 | Loco10 | Loco11 | Loco12 | Loco13 | Loco14 | HWE |
| --- | --- | --- | --- | --- | --- | --- | --- | --- | --- | --- | --- | --- | --- | --- | --- | --- |
| EV | Yellow | 0.58 | 0.169 | **0.00** | **0.00** | 0.85 | 0.35 | **0.00** | **0.01** | **0.01** | **0.01** | **0.01** | 0.51 | **0.01** | 0.57 | 43% |
| BS | Yellow | **0.00** | **0.00** | 0.92 | **0.00** | **0.00** | 0.21 | **0.00** | **0.00** | **0.00** | **0.00** | **0.00** | **0.00** | **0.00** | **0.00** | 14% |
| BR | Red | **0.00** | **0.00** | **0.05** | **0.00** | **0.03** | **0.00** | 0.09 | **0.00** | 0.46 | **0.01** | **0.00** | 0.77 | **0.00** | **0.00** | 21% |
| AE | Red | **0.01** | **0.00** | **0.00** | **0.00** | **0.00** | **0.00** | 0.27 | **0.00** | **0.00** | 0.37 | 0.21 | **0.00** | 0.81 | **0.00** | 29% |
| EU | Red | **0.00** | **0.00** | **0.03** | **0.00** | 0.75 | 0.32 | **0.00** | 0.64 | **0.00** | **0.00** | **0.01** | 0.96 | **0.00** | 0.76 | 36% |
| ST | Green | **0.03** | 0.21 | **0.05** | **0.00** | **0.00** | **0.00** | **0.02** | **0.00** | **0.00** | **0.00** | 0.14 | 0.06 | **0.00** | 0.12 | 29% |
| BN | Green | 0.52 | **0.01** | 0.91 | **0.04** | **0.00** | **0.00** | 0.11 | 0.46 | 0.83 | 0.14 | 0.85 | **0.00** | **0.00** | **0.02** | 50% |
| RE | Green | 0.24 | 0.38 | 0.29 | **0.00** | **0.00** | 0.97 | 0.11 | 0.99 | 0.85 | **0.00** | **0.01** | **0.00** | 0.94 | 0.15 | 64% |

Abbreviations: *EV = Private Reserve of Natural Patrimony Estação Veracel; BS = Boa Sorte Farm; BR = Una Biological Reserve; AE = Farm Alto da Esperança; EU = Ecoparque de Una; ST = Private Reserve of Natural Patrimony Serra do Teimoso; BN = Brasilia National Park; RE = Roncador Ecological Reserve; HWE = Percentagem of loci in Hardy-Weinberg Equilibrium. P values Significant are shown in bold.

**
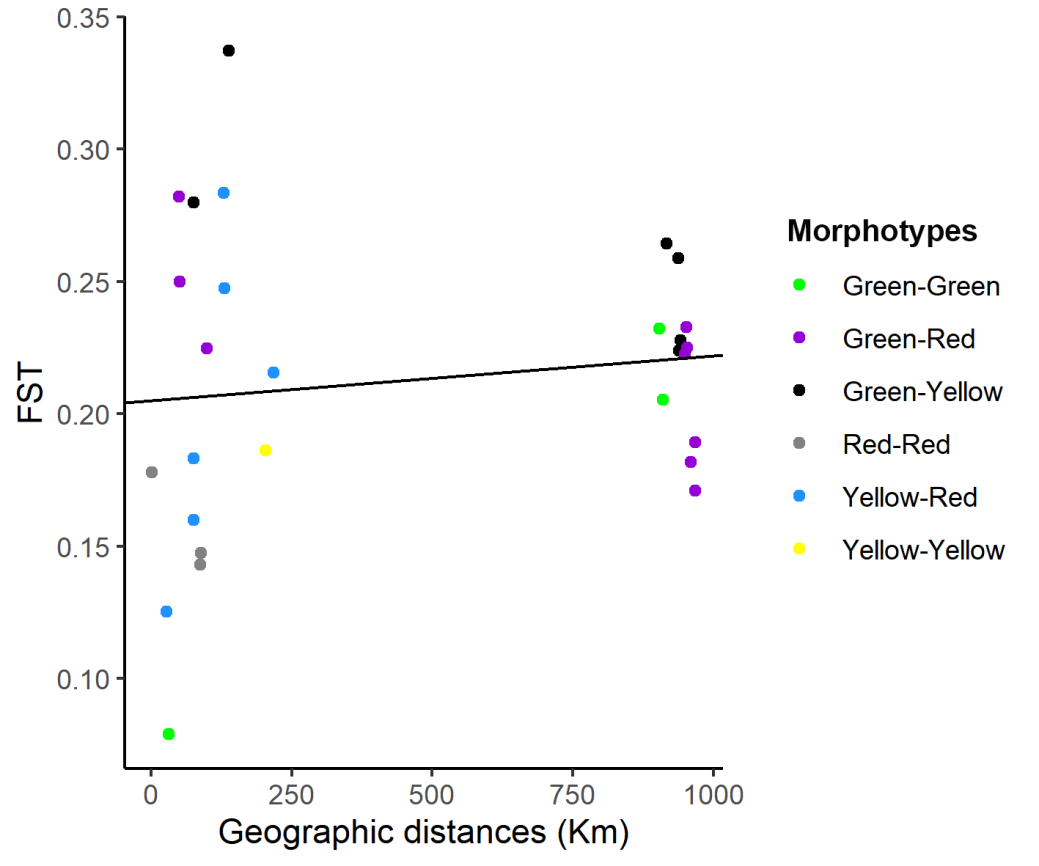
**

**Figure S1.** Relationship between pairwise F_ST_ and geographic distances for the eight populations sampled of *E. edulis* and their respective morphotypes, with Mantel test (r=0.134, p=0.33).


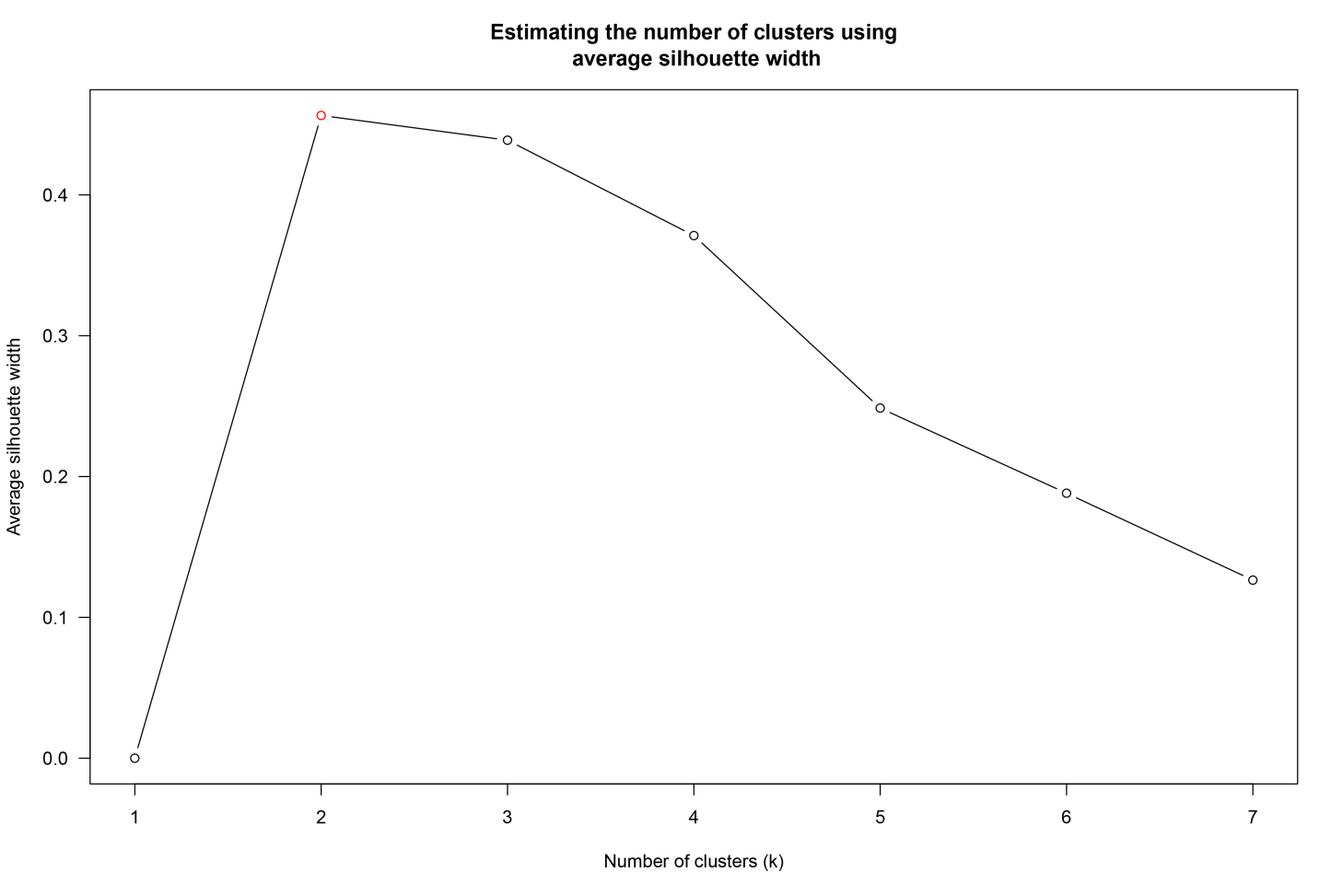


**Figure S2.** Estimated number of groups using the average method for the number of populations K in 250 individuals of *E. edulis* of the eight populations sampled.


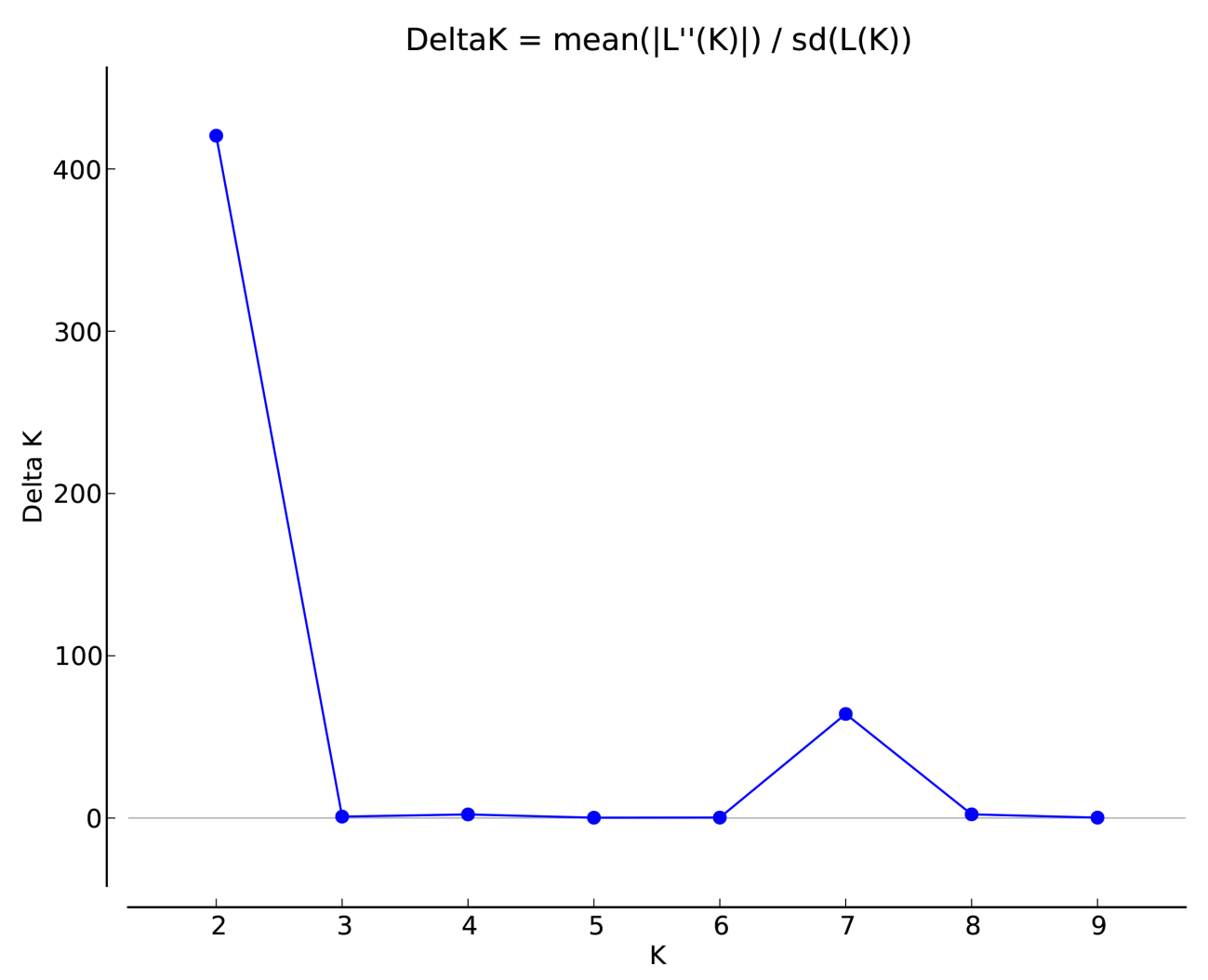


**Figure S3.** Variation of the second order of the average values ​​of maximum likelihood for the number of populations K in 250 individuals of *E. edulis* of the eight populations sampled.
